# COVID-19 infection control practices in designated quarantine hotels in Hong Kong Special Administrative Region SAR (China), 2020–2022: key elements in preparing for the next pandemic

**DOI:** 10.5365/wpsar.2025.16.1.1167

**Published:** 2025-03-04

**Authors:** Edmond Siu-keung, Hong Chen, Shuk Kwan Chuang

**Affiliations:** a Infection Control Branch, Centre for Health Protection, Department of Health, Hong Kong Special Administrative Region, China.; b Surveillance and Epidemiology Branch, Centre for Health Protection, Department of Health, Hong Kong Special Administrative Region, China.

## Abstract

**Problem:**

Despite the widespread use of designated quarantine hotels to minimize the transmission of COVID-19 from imported cases, there is scant literature on the infrastructure and operational requirements of such facilities.

**Context:**

Travellers to Hong Kong Special Administrative Region (SAR) (China) were required to undergo quarantine in designated hotels for up to 21 days. Prior to operation, all these hotels were modified and hotel staff received structured training in infection control practices.

**Action:**

We conducted retrospective reviews of the procedures and operational protocols that were followed to convert and manage commercial hotels as quarantine hotels during the early part of the pandemic. We also reviewed the training provided and compliance monitoring. Finally, we reviewed intra-hotel outbreak investigations that were conducted between April 2021 and June 2022.

**Outcome:**

Designated quarantine hotels received 842 510 quarantined travellers from December 2020 to October 2022. Ten outbreaks were reported, affecting 28 guests (0.003%) and two staff. Prompt epidemiological investigation and action stopped further transmission.

**Discussion:**

In Hong Kong Special Administrative Region SAR (China), designated quarantine hotels successfully minimized COVID-19 transmission from imported cases to the community and should be considered as part of integrated response plans for future pandemics. Based on our COVID-19 pandemic experience, we recommend specifying requirements for quarantine centres and hotels to ensure adequate ventilation inside guest rooms and corridors, functioning drainage systems and the adoption of stringent infection control practices. We also recommend the installation of closed-circuit television cameras in all common areas to support compliance monitoring and outbreak investigation.

## PROBLEM

During rapidly evolving infectious disease epidemics like the recent COVID-19 pandemic, the rapid scaling up of designated quarantine hotels (DQHs) proved crucial in preventing community transmission from imported cases. However, at the time of the pandemic onset, there were no international standards governing the infrastructural or operational requirements of quarantine hotels. While the World Health Organization (WHO) regularly issued guidance on infection control throughout the pandemic period – covering topics such as hygiene practices, the use of masks and waste management – the guidance focused on preventing transmission in health-care facilities and the community and was not specifically tailored to quarantine hotels. (*1*, *2*) Post-pandemic, there has also been a notable lack of literature documenting public health practices on the preparation and operation of quarantine hotels. This knowledge gap is concerning, given the critical role played by DQHs in preventing the transmission of COVID-19 in hotels and to the local community. We addressed this gap by reviewing the operation of DQHs in  Hong Kong Special Administrative Region (SAR) (China) during the period December 2020 to October 2022. We hope that the lessons identified by this review will inform strategies for improving DQH management in preparation for future pandemics.

## CONTEXT

Following WHO’s declaration of COVID-19 as a public health emergency of international concern on 30 January 2020, (*3*) many countries including Hong Kong Special Administrative Region SAR (China) used quarantine hotels to delay transmission to the community. (*4*-*7*) Their use is a recognized public health containment measure to slow down community transmission from imported cases by identifying and isolating individuals, thereby buying time to implement other response measures and to build up population immunity through vaccination.

In Hong Kong Special Administrative Region SAR (China), travellers were required to undergo quarantine in designated centres or hotels for up to 21 days during different periods of the pandemic (**Table 1**). Additional measures were introduced to reduce the risk of transmission, including:

**Table 1:** Quarantine measures in Hong Kong Special Administrative Region SAR (China), 8 February 2020 to 26 September 2022

Date	Quarantine measures
**8 February 2020**	**Persons returning from China were required to undergo home quarantine for 14 days.**
**1 March 2020**	**Inbound travellers arriving from specific high-risk overseas areas in the previous 14 days were required to stay in quarantine centres.**
**14 March 2020**	**Inbound travellers arriving from specific high-risk overseas areas in the previous 14 days were required to undergo compulsory home quarantine.**
**11 May 2020**	**Inbound travellers arriving from additional high-risk areas were required to stay in quarantine centres for 14 days.**
**25 July 2020**	**Inbound travellers arriving from additional high-risk areas were required to quarantine in hotels for 14 days.**
**25 December 2020**	**All inbound travellers were required to quarantine in DQHs for 21 days.**
**5 February 2022**	**All inbound travellers were required to quarantine in DQHs for 14 days.**
**1 April 2022**	**All inbound travellers were required to quarantine in DQHs for 7 days, if they had been vaccinated twice.**
**12 August 2022**	**Travellers were required to quarantine in DQHs for 3 days, followed by medical surveillance for 4 days.**
**26 September 2022**	**No quarantine was required at DQHs, but travellers had to undergo medical surveillance for 3 days followed by a 4-day self-monitoring period.**

DQH: designated quarantine hotel.

mandatory use of face masks in public areas;
school suspension;
teleworking for civil servants;
restrictions on restaurants’ opening hours;
temporary closure of community facilities such as sports centres, libraries, karaoke lounges, bars and cinemas; and
physical distancing measures.

**Fig. 1:**
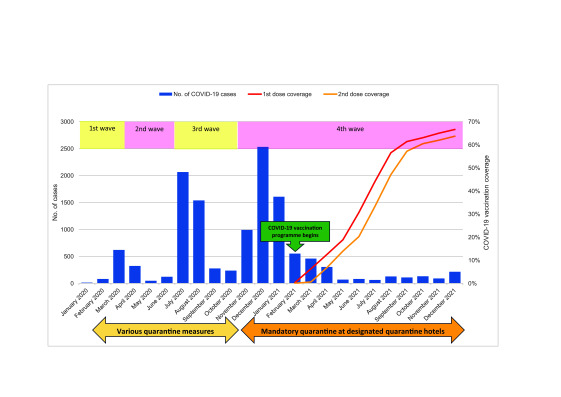
COVID-19 cases and vaccination coverage before and during the operation of designated quarantine hotels in Hong Kong SAR (China), January 2020 to December 2021

COVID-19 vaccination of the population was introduced on 26 February 2021. The pandemic in Hong Kong Special Administrative Region SAR (China) consisted of five waves, resulting in a total of 1 745 505 cases and 10 116 deaths (**Table 2**; **Fig. 1**). (*7*)

[**insert** Figure 1]

**Table 2:** Number of cases and deaths during COVID-19 waves in Hong Kong Special Administrative Region SAR (China), 23 January 2020 to  25 September 2022

COVID-19 wave	Period	No. of cases	No. of deaths
**1st**	**23 January to 14 March 2020**	**142**	**4**
**2nd**	**15 March to 30 June 2020**	**1064**	**4**
**3rd**	**1 July to 31 October 2020**	**4118**	**103**
**4th**	**1 November 2020 to 30 April 2021**	**6451**	**101**
**Window phase**	**1 May to 30 December 2021**	**861**	**1**
**5th**	**31 December 2021 to 25 September 2022**	**1 732 869**	**10 116**

*Source*: Wong et al. (*7*)

Notably, most quarantine hotels globally were not purpose-built quarantine facilities, but rather commercial complexes that were adapted for quarantine use. Intra-hotel transmission of SARS-CoV-2 was reported in several countries and areas, including Australia, China, New Zealand, Spain, China, Taiwan (China) and Thailand. (*4*, *5*, *8*-*12*) In the case of the facility in New Zealand, (*9*) detailed investigation revealed that transmission may have occurred due to brief periods of simultaneous door-opening, which may have caused airborne infectious particles to disperse down a concentration gradient, across the corridor and into the confinees’ rooms.

## ACTION

As part of a designated team set up by the Government to oversee the management of DQHs, we retrospectively reviewed the protocols and procedures that were followed during the pandemic to (i) select and convert hotels to DQHs, (ii) operate the chosen hotels as DQHs and (iii) investigate any intra-hotel outbreaks. We also reviewed available epidemiological information on the number of cases and vaccination coverage from the Centre of Health Protection at the Department of Health.

### Transforming ordinary hotels into designated quarantine hotels

Available WHO guidelines on infection control in the community (*1*, *2*) were consulted in drawing up the operational rules and protocols for setting up and operating DQHs. A multidisciplinary team comprising public health physicians, infection control personnel and government engineers inspected all potential hotels, conducting a thorough on-site assessment of the infrastructure, operations and staff composition to ascertain feasibility. The team also advised on the modifications required to prevent intra-hotel transmission. Inspections covered: reception areas; use of designated lifts and routes from the reception area to guest rooms; meal arrangements; linen and waste management; the setup inside guest rooms (for example, simple furniture and bed linen covered with materials for easy disinfection, the provision of disposable water bottles, plastic bags for waste disposal); designated routes for transferring sick or positive cases to hospitals; clean routes for confinees to leave hotels after completion of their quarantine period; and the setup of closed-circuit television (CCTV) cameras in reception areas, guest floors, public areas and back staircases for monitoring compliance. Mini posters were displayed in prominent sites inside guest rooms as a reminder for confinees to wear a face mask before opening doors and to pour 500 mL of water into each drain outlet (U-trap) once a week to prevent vertical transmission through the drainage system. Special attention was paid to the adequacy of the ventilation systems: hotels had to have negative room pressure (to ensure airflow from the corridor to the guest rooms), toilet exhaust fans with a flow rate of > 18 L/s and an adequate distance (> 7.5 m) from exhaust fans in “dirty” zones to fresh air intake of “clean” zones (to minimize the risk of transmission to nearby residential buildings).

For hotels considered suitable to serve as DQHs, infection control personnel provided training to the hotel staff on the donning and doffing of personal protective equipment (PPE), proper hand hygiene at critical moments, environmental cleaning and disinfection using hypochlorite, and the handling of sick patients and potentially contaminated waste. The PPE required for different staff was fully explained based on risk assessment, including the wearing of respirators for those working in dirty zones.

### Operation

Confinees were required to follow the designated path to their room at the beginning of their quarantine period, which included the use of a designated lift. During the quarantine period, confinees were not allowed to leave their rooms and visitors were not permitted. Infection prevention measures were taken around meal provision, handling of clean and dirty linen and clothing, and waste management. The hotel staff were advised to place meals and other items on a chair or table outside guest room doors, and to clean and disinfect the area regularly. Conversely, confinees were instructed to put their used and soiled items and waste in waterproof plastic bags and leave them outside their doors. A designated trolley was used for transporting the laundry and waste bags in assigned “dirty” lifts to a designated area for temporary storage before being transported outside for further management. Donning and doffing areas for PPE were set up to ensure adequate protection of the staff in the daily handling of meals, linens and waste.

Trained health-care workers took nasopharyngeal swabs from all confinees near the guest room doors for SARS-CoV-2 testing by polymerase chain reaction (PCR). Portable high-efficiency particulate air (HEPA) filters were used to minimize droplet spread during specimen collection. Persons who tested positive were immediately transferred to hospitals for isolation and treatment. Close contacts who were staying in the same room were transferred to purpose-built quarantine facilities (non-DQH quarantine facilities) for the continuation of their quarantine. Approved cleaning companies cleaned and disinfected the affected rooms with real-time monitoring of the whole process through CCTV cameras by the hotel staff or nurses on the compliance team to ensure proper cleaning and disinfection.

The compliance team for infection control comprised a public health physician and over 40 nurses trained in infection control. The team conducted daily on-site inspections of the DQHs to monitor infection control practices. In case of non-compliance of COVID-19 regulations, confinees might be subject to verbal or written warnings or legal liability based on the severity of the infraction. Another team comprising members of the disciplinary services were retired police officers who helped ensure that the confinees stayed in their rooms.

### Outbreak investigation and control

When someone in quarantine became ill or tested positive for COVID-19, they were sent to hospital for treatment. Positive PCR specimens underwent whole-genome sequencing at a public health laboratory. If more than one case at the same DQH had the same or a highly similar genetic sequence, this was interpreted as indicating intra-DQH transmission.

When there was a suspected outbreak of COVID-19 within a DQH, prompt investigation was carried out by a multidisciplinary team comprising epidemiologists, infection control specialists, clinical microbiologists, engineers and technicians for inspection of drainage systems. Epidemiologists interviewed the DQH staff and reviewed CCTV camera footage to assess for possible interaction between cases or lapses in infection control measures. A smoke test was performed to test the airflow direction between the guest rooms and corridors. Environmental swabs were taken to identify potential fomite contamination in different areas of the hotel to inform possible routes of transmission. Where necessary, prompt action was implemented to minimize the risk of further spread within the DQH.

## OUTCOME

The number of DQHs in operation from December 2020 to October 2022 ranged from 30 to 68. A total of 842 510 inbound travellers underwent mandatory quarantine in DQHs. By ensuring early identification and isolation, the use of DQHs successfully delayed transmission from imported cases to local communities. This important containment measure provided an opportunity to put in place other response measures, and for the community to build up immunity through vaccination. COVID-19 vaccines became available in Hong Kong Special Administrative Region SAR (China) in February 2021, and the coverage climbed to over 60% for first and second doses by December 2021 (**Fig. 1**).

Significantly, intra-hotel transmission was minimal: a total of 10 clusters were reported, involving 28 guests (0.003% of all guests) and two staff (**Table 3**). The number of cases affected in each cluster ranged from two to six. The reason for these clusters was attributed to either inadequate infrastructure (such as poor ventilation systems in guest rooms or stagnant air in the corridors), improper infection control practices (for example, the use of an inappropriate mask with a valve) or non-compliance with environmental disinfection procedures (**Table 3**).

**Table 3:** Factors possibly contributing to intra-hotel transmission of COVID-19 occurring in designated quarantine hotels in Hong Kong Special Administrative Region SAR (China), 17 April 2021 to 1 June 2022

Hotel	Date of detection	No. of persons affected	Likely contributing factors
**A**	**17 April 2021**	**3 confinees**	**Improper handling of meal delivery**
**B**	**23 April 2021**	**4 confinees**	**When the guest room window and door were both open, air spread from the index case’s room through the corridor to other guest rooms.**
**C**	**2 July 2021**	**1 confinee, ** **1 cleaning staff**	**Non-compliance with infection control practices by the cleaning staff when disinfecting the index case’s guest room**
**D**	**17 August 2021**	**3 confinees**	**When the guest room window and door were both open, air spread from the index case’s guest room through the corridor to other guest rooms.**
**E**	**11 November 2021**	**2 confinees**	**Index case engaged in vigorous exercise; no mask wearing when opening the guest room door; inadequate environmental cleaning and disinfection**
**F**	**22 November 2021**	**2 confinees**	**Index case wore a mask with a valve; no mask wearing when opening the guest room door; inadequate negative pressure inside the guest room**
**G**	**16 January 2022**	**6 confinees**	**Inadequate negative pressure inside the guest room; poor ventilation in the corridor leading to the guest room**
**H**	**20 May 2022**	**4 confinees**	**Fire exit door was open when specimens were taken from confinees, which may have led to transmission between two floors.**
**I**	**27 May 2022**	**3 confinees**	**Air spread from the index case’s guest room to the corridor; no mask wearing when opening the guest room door**
**J**	**10 June 2022**	**1 staff**	**The ventilation outlet of a dirty area was too close to the inlet of a clean zone (staff area).**

## Discussion

During their operation, DQHs minimized the spillover from imported cases to the community despite a high level of COVID-19 infection around the world, until Hong Kong Special Administrative Region SAR (China) was hit by a fifth wave of the highly transmissible Omicron variant in January 2022. Thorough preparation of DQHs, training of staff in infection control practices and prompt intra-hotel outbreak investigation were among the factors that contributed to the effectiveness of DQHs in reducing transmission of SARS-CoV-2 from imported cases.

In terms of preparing DQHs, particular attention was paid to ventilation systems, given that studies conducted in other quarantine facilities, such as in New Zealand and China, Taiwan (China), had identified inadequate ventilation systems, simultaneous door-opening and interaction between positive cases as risk factors for hotel outbreaks. (*5*, *9*, *10*) Subsequent studies have found that the number of exhaust fans and their distance from occupants, ventilation rates and indoor airflow patterns were critical elements in preventing indoor transmission in quarantine facilities. (*13*-*15*) A simulation study in a quarantine hotel demonstrated that SARS-CoV-2 could be transmitted from air inside a guest room to the corridor outside, and then to other rooms on the same floor. (*15*)

During operation, prompt epidemiological investigation of outbreaks followed by swift action were shown to be effective in minimizing the number of cases affected in intra-hotel outbreaks, with six confinees making up the largest cluster. Moreover, only two hotel staff were infected in the reported clusters. No member of the swabbing team was infected. Outbreak investigation and action was facilitated by regular SARS-CoV-2 testing and genetic analysis of the isolated viruses. Immediate removal of positive cases and close contacts to designated isolation or quarantine facilities stopped further transmission. Remedial actions, including the installation of air purifiers in hotel rooms and corridors, ensuring confinees wore proper masks before opening their door, and proper disinfection of guest rooms and common areas, were implemented after each outbreak to address the possible contributing factors. Continuous review and monitoring of infection control practices by the compliance team also helped to ensure that staff were protected from infection. (*16*) Lessons learned were shared to prevent the occurrence of similar cases in other DQHs.

The successful preparation and operation of DQHs owed much to the joint efforts of health-care and other workers, including engineers and retired members of the disciplinary forces. As the understanding of the route of transmission and infectivity of SARS-CoV-2 evolved, infection control personnel worked closely with engineers in implementing evidence-based advice on ventilation, for example, the installation of air purifiers at strategic locations in the hotels and modifications to ventilation systems to improve air change.

Our findings and lessons learned can be applied to other countries and areas in the Western Pacific Region and other parts of the globe that use hotels for quarantine purposes to prevent the importation of SARS-CoV-2. While hotel facilities may vary, the general principles of infection control can be universally applied. One of the challenges to implementing stringent infection control measures is the recruitment of health-care professionals for compliance monitoring. During the pandemic, there was high demand for health-care staff in various facilities, including not only hospitals but also quarantine centres and vaccination centres. We successfully recruited retired nurses, including those with knowledge and skills in infection control, to meet operational needs. Similarly, we were able to call upon retired members of the disciplinary forces to assist in ensuring that confinees observed the regulations.

Limitations of the current review include the possible underreporting of COVID-19 cases, especially in the later phase of the pandemic when the length of time travellers were required to stay in DQHs was reduced to 7 days. However, confinees were required to report to community centres for testing for a further 14-day period after leaving a DQH. This should have reduced the likelihood of underreporting. Second, some cases of intra-hotel transmission might have been missed. Third, recall bias among cases and hotel staff might have affected the epidemiological investigations; however, such information was verified by hotel records and CCTV camera footage as far as possible.

With the gradual return to normalcy, WHO has urged countries to prepare for “disease X” and form integrated plans for responding to any respiratory pathogen including influenza and coronaviruses. (*17*) If quarantine hotels are included in containment measures to delay the importation of an emerging infectious pathogen, it is crucial to reflect on the experience gained from their use during the COVID-19 pandemic. (*18*) Based on our experience, we recommend the adoption of infrastructure requirements for DQHs to ensure that adequate ventilation, air purification and drainage systems are installed before operation. We also recommend the installation of CCTV cameras in all common areas to support the monitoring of compliance with infection control practices (e.g. mask wearing and surface disinfection) and outbreak investigation. These measures will help countries formulate a better plan to tackle the next pandemic.
